# An Uncommon Association: Eltrombopag and Autoimmune Hepatitis

**DOI:** 10.1155/crhe/6637055

**Published:** 2025-04-01

**Authors:** Fady Salama, Hassnain Syed, Nimish Thakral

**Affiliations:** Department of Digestive Disease and Nutrition, University of Kentucky, Lexington, Kentucky, USA

## Abstract

Autoimmune hepatitis (AIH) is an immunological disorder of the liver characterized by hepatic necroinflammation due to the break in self-tolerance to autoantigens. Many medications have been linked to drug induced AIH. A review of the literature reveals no documented association between Eltrombopag and AIH. Eltrombopag is a thrombopoietin receptor agonist used to help manage thrombocytopenia in immune mediated thrombocytopenia and chronic liver disease. Here, we present a case of a 59-year-old female with refractory thrombocytopenia who was started on Eltrombopag, and later developed AIH.

## 1. Case Presentation

Our patient is a 59-year-old female with history of morbid obesity. In 1/2020, patient presented to the ER with epistaxis and was found to have a platelet level of 26 k/uL, with nadir of 15 k/uL, despite receiving platelet transfusions. Extensive workup, including a bone marrow biopsy was negative. While being worked up for thrombocytopenia by hematology, she was found to have splenomegaly, abdominal ultrasound revealed cirrhosis with splenomegaly and subsequent CT abdomen with contrast showed lobulated liver with signs of portal hypertension like splenomegaly and extensive varices. Diagnosis of NASH cirrhosis was based on risk factors like obesity (BMI 42 kg/m^2^ at time of diagnosis) and hypothyroidism. No liver biopsy was done at that time since transaminases were normal, smooth muscle antibody was negative, viral hepatitis showed positive HCV antibody but undetected HCV PCR. Multidisciplinary team consensus concluded a combination of cirrhosis and immune mediated thrombocytopenia as the etiology of thrombocytopenia, and she was started on Eltrombopag 50 mg PO daily. Platelets count was as low as 20 k/uL before starting Eltrombopag.

**Figure 1:**
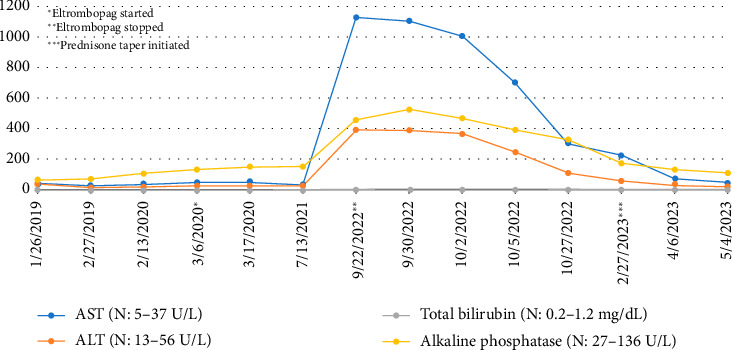
Liver chemistry trend upon eltrombopag initiation and cessation.

Patient showed dramatic improvement in her platelet count after starting Eltrombopag, reaching levels within normal range (150 k/uL–450 k/uL). She continued to follow Hepatology for cirrhosis management. About 2 years after starting Eltrombopag, the patient had a significant elevation in liver chemistries: AST 1132 U/L, ALT 393 U/L, total bilirubin (TB) 4.4 mg/dL, alkaline phosphatase (ALP) 460 U/L, and INR 1.3 (Figure 1). The patient's only symptom was jaundice. She denied any new medications or supplements. Infectious workup including viral hepatitis panel CMV, EBV, and HSV PCR were negative. Acetaminophen level was normal (Table 1). Initial thought was that patient developed portal vein thrombosis as a complication of Eltrombopag, however liver doppler showed patent portal vein. Immunological workup revealed normal antinuclear antibody titer, normal antimitochondrial antibody level, and strongly positive antismooth muscle antibody level at 1:2560 which was negative on initial workup for cirrhosis. Immunoglobulin G (IgG) level was elevated at 1835 mg/dL (n: 720–1589 mg/dL). The other long-term medications she was taking were levothyroxine and sertraline. The patient was instructed to immediately stop the Eltrombopag. One week later, LFTs were rechecked which showed continued high transaminases, although stable, but worsening TB at 7.1 mg/dL, ALP at 528 U/L, and INR of 1.4.

**Figure 2:**
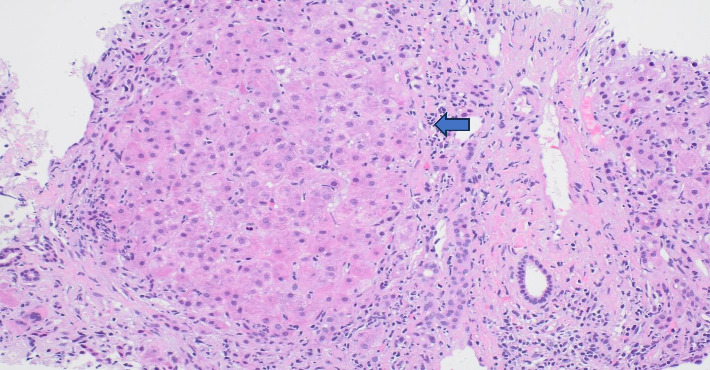
Liver biopsy histology. Inflammation, mainly lymphocytic (arrow), but with occasional plasma cells.

A liver biopsy was obtained and showed stage 3-4 fibrosis, interface hepatitis with predominant lobular involvement and the portal areas showing lymphocytic infiltration with occasional plasma cells. There was no evidence of ductopenia or periductal fibrosis. No granulomas were seen. The lobule examined was negative for fatty change or ballooning degeneration (Figure 2).

The patient was diagnosed with drug-induced autoimmune hepatitis (DIAIH) from Eltrombopag. There was an initial improvement in her LFTs 1-2-month postcessation of the drug, however transaminases remained elevated. Patient was subsequently initiated on a prednisone taper with normalization of the transaminases within 2 months.

## 2. Discussion

DIAIH resembles classic idiopathic AIH but usually resolves upon cessation of the offending agent. Furthermore, advanced fibrosis is seldom seen with DIAIH. There are several medications that have been linked to DIAIH. Diagnosis of DIAIH can be very challenging and difficult to distinguish from idiopathic AIH as it presents like idiopathic AIH in regard to clinical presentation, serological and histological features. Drugs like infliximab, hydralazine, minocycline, and methyldopa are common examples [1].

DIAIH is more common in females and usually present acutely, can be associated with hypersensitivity symptoms like fever, rash, and eosinophilia in 30% of cases [2].

Our patient did have cirrhosis, but this was diagnosed prior to initiation of Eltrombopag. Furthermore, her body mass index of 42 kg/m^2^ suggests NAFLD-related etiology of cirrhosis. The liver biopsy result did not show any evidence of fatty liver, which could be explained by burnt out NASH in the setting of cirrhosis. Another less likely possibility that patient could have had AIH and was in remission since liver biopsy showed no signs of NASH.

Any DI liver injury (DILI) is a diagnosis of exclusion. After ruling out other etiologies of the patient's deranged LFTs, including a full assessment of medications history, we suspected DILI from Eltrombopag as the inciting factor [3, 4].

In view of this case, clinicians should strongly consider checking LFTs prior to treatment initiation and during treatment with Eltrombopag. Eltrombopag should be discontinued if ALT levels increase to greater than or equal to 3x upper level of normal (ULN) in patients with normal LFTs prior to treatment.

Interestingly, the time to onset of DIAIH is usually more than 6 months and can be up to several years after initiation of therapy. In some situations, onset is more rapid, and the clinical phenotype resembles acute hepatitis, but onset with appearance of autoantibodies is rare before 2 months [5].

Our patient fits this criterion, with biopsy proven DIAIH. A review of the literature, including a review of major guidelines, does not reveal any cases of DIAH caused by Eltrombopag. This case highlights a potentially major sequela of Eltrombopag that should be considered in the event of elevated transaminases seen in patients taking Eltrombopag.

## Figures and Tables

**Table 1:** Elements assessed during work up of DILI.

Elements assessed	Comments
Name of the medication with dose and regimen	Eltrombopag 50 mg PO daily
Date that the medication was started	3/6/2020
Date the medication was stopped	9/22/2022
Symptoms and time of onset	New onset of jaundice reported on 9/22/2022; no other symptoms. AST 1132, ALT 393, total bilirubin 4.4, ALP 460, PT 15.3 s, INR 1.3
Evidence of clinical and biochemical recovery after discontinuation of the implicated drug	After drug cessation, notable decline in elevated liver chemistries although still notable elevation seen in transaminases. Therefore, prednisone taper initiated on 3/6/2023 with normalization of transaminase levels within 2 months
IgM anti-HAV	Negative
HBsAg; IgM anti-HBc	Negative; negative
Anti-HCV	Negative
Antinuclear antibody	< 1:80 (N: < 1:80)
Globulin level	IgG 1835 (N: 720–1589 mg/dL)
Ultrasound of the liver with Doppler	Cirrhotic liver morphology with no suspicious lesions; no ascites; patent portal vein with antegrade flow; patent hepatic vasculature; unremarkable gallbladder; common bile duct: 5 mm
Antismooth muscle antibody	1: 2560 (N: < 1:20)
CMV, EBV, HSV PCR	Not detected
Liver biopsy histology	Bridging fibrosis (stage 3-4), interface hepatitis with predominant lobular involvement and the portal areas showing lymphocytic infiltration with occasional plasma cells

## Data Availability

The manuscript data used to support the findings of this study are available from the corresponding author upon request.
